# Survival and predictors of death in people with HIV-associated lymphoma compared to those with a diagnosis of lymphoma in general population

**DOI:** 10.1371/journal.pone.0186549

**Published:** 2017-10-31

**Authors:** Antonella Cingolani, Alessandro Cozzi Lepri, Luciana Teofili, Laura Galli, Valentina Mazzotta, Gian Maria Baldin, Stefan Hohaus, Alessandra Bandera, Lucia Alba, Nadia Galizzi, Antonella Castagna, Antonella D'arminio Monforte, Andrea Antinori, on behalf of ICONA Foundation Study group

**Affiliations:** 1 Infectious Diseases Fondazione Policlinico Universitario A. Gemelli-Università Cattolica S. Cuore, Roma, Italy; 2 Centre for Clinical Research, Epidemiology, Modelling and Evaluation (CREME), Institute for Global Health, University College London, London, United Kingdom; 3 Hematology, Fondazione Policlinico Universitario A. Gemelli-Università Cattolica S. Cuore, Roma, Italy; 4 Infectious Diseases, San Raffaele Scientific Institute, Milano, Italy; 5 National Institute for Infectious Diseases L. Spallanzani, Roma, Italy; 6 Infectious Diseases San Gerardo Hospital, Monza, Italy; 7 San Paolo Hospital, University of Milano, Milano, Italy; University of New South Wales, AUSTRALIA

## Abstract

**Objectives:**

to compare overall survival in HIV-associated lymphoma (HIV-L) and lymphoma raising in HIV-negative population (nHIV-L) and to identify predictors of increased risk of death.

**Methods:**

All HIV+ patients with HIV-associated lymphoma (Hodgkin lymphoma, HL; non-Hodgkin Lymphoma, NHL) observed between 1.2000 and 12.2013 in the ICONA Foundation Study cohort or in three collaborating centres, and, as control group, nHIV-L individuals followed in one of the four collaborating centres over the same time period, were included. Survival estimates were calculated by use of Kaplan-Meier (KM) and multivariable Cox regression models.

**Results:**

1,331 pts were included (465 HIV-L, 866 nHIV-L): 909 (68%) NHL, 422 (32%) HL. 3 years-cumulative probability (95% confidence interval, CI) of death was higher in HIV-L compared to nHIV-L in NHL (38% (33–44) vs. 22% (19–26); p<0.001), and HL (22% [15–29] vs. 10% (6–13), p<0.001). Among HL, HIV was associated with an increased risk of death (hazard ratio [HR] = 2.37 [95% CI: 1.24–4.55], p = 0.009) independently of calendar year, age, gender, type of chemotherapy and stage; in NHL, HIV was no longer an independent predictor of death after controlling for rituximab use and IPI (HR = 1.26 (0.97–1.63), p = 0.08).

**Conclusions:**

Our analysis shows a reduced overall survival in HIV+ patients diagnosed with lymphoma compared to HIV-negative controls. Whereas in HIV people with HL, the increased risk of death was confirmed even after adjustment for main confounders, the association between HIV status and survival in NHL appears to be somewhat attenuated after controlling for more aggressive presentation and lower frequency of rituximab use in HIV-+ people.

## Introduction

Since the introduction of combined antiretroviral therapy (cART), survival of HIV-associated lymphoma (HIV-L, Hodgkin lymphoma [HL] and non-Hodgkin [NHL]) has considerably improved, due to increased response to chemotherapy in people taking cART [1–4]. Also the incidence of HIV-related lymphoma has significantly declined since the introduction of cART but this condition still represents one of the most prevalent causes of hospitalization occurring in HIV-infected patients even in the cART era [5] and a major cause of morbidity and mortality [6]. The improved prognosis seen in recent years is likely to be due both to the use of more intensive chemotherapy regimens, similar to those used for lymphoma raising in HIV-negative patients, and to increased immune recovery and better control of HIV infection itself due to the availability of more potent antiretroviral regimens. Nevertheless, data comparing clinical outcome in HIV-related lymphomas with those occurring in the general population are still limited and controversial. More aggressive clinical and histologic features of HIV-related compared to HIV- unrelated lymphomas were reported as possible determinants of a worse prognosis in diffuse large B cell lymphoma (DLBCL) [7]. A more conservative treatment approach with limited access to standard chemotherapy regimens were reported as detrimental both for DLBCL and Hodgkin lymphoma (HL) [8–9].

The objectives of this analysis were to compare the overall survival of HIV-associated lymphoma with those occurring in a suitable control group of HIV-negative individuals included from one of the participating clinical sites and to identify other factors besides HIV-status that are associated with the risk of a reduced survival.

## Methods

All patients in the Italian Cohort On aNtiretroviral naïve (ICONA) Foundation cohort or in three collaborating clinical sites were included with a diagnosis of HIV-L (NHL and HL) observed between January 1, 2000 and December 31, 2013 were included. ICONA Foundation Cohort is an observational cohort of HIV-infected individuals who are antiretroviral naïve at the time of enrolment [10]. This cohort was set up in January, 1997 and to date consists of more than 12,000 patients from 50 infectious disease units in Italy. Initiation and discontinuation dates of each antiretroviral drug, HIV-viral load and CD4 cell count at each clinical visit (every 4–6 months on an average), together with clinical HIV- and non-HIV related events, were recorded for each enrolled patient. As the ICONA Foundation cohort only includes HIV-infected patients who are antiretroviral-naïve at enrolment, in order to increase the sample size all HIV-infected individuals with a diagnosis of lymphoma which occurred over the same time window (Jan 2000-Dec 2013) in four of the largest clinical sites contributing patients to ICONA (UCSC, San Raffaele, INMI, San Gerardo) were also included in the analysis. Similar data are recorded in the databases of these sites which have been harmonised according to a specific standard operating procedure (SOP) and merged with those of ICONA. In addition, for this project a specific questionnaire has been developed for people with a diagnosis of HIV-L. Specifically, the following data related to neoplasia were retrospectively collected for each patient diagnosed with a lymphoma: date of lymphoma diagnosis, histotype, Ann Arbor stage, regimens of chemotherapy, use of rituximab, radiotherapy, date of last observation and the cause of death.

All controls were HIV-negative patients (nHIV-L) consecutively diagnosed with lymphoma over the same calendar period at the general hematology unit of one of the four described large Icona sites (UCSC). This is a reference center for diagnosis and cure of lymphoma in the north of Rome. The same exact questionnaire for lymphoma-related items has been used to retrospectively collect data in this control group.

All individuals signed an informed consent form prior to enrolment and the study was approved by the Ethics Committee of each participating institution listed in the Acknowledgments.

### Statistical analysis

Study population was described and main characteristics at the time of diagnoses of lymphoma compared between HIV-L and nHIV-L using chi-square test for categorical factors and non-parametric tests comparing median values for continuous variables.

The effect of HIV-status on overall survival (OS) was assessed using standard survival analysis techniques. Participants’ follow-up time accrued from the date of the diagnosis of lymphoma up to the date of death or last clinical visit at which the person was seen alive.

Survival estimates were calculated using Kaplan-Meier (KM) curves and predictors of OS identified by Cox regression modelling. Unadjusted and adjusted hazard ratio (HR) after controlling for a number of potential confounding factors (calendar year of diagnosis, age, gender, International Prognostic Index score and use of rituximab for NHL or ABVD for HD lymphomas) were calculated and tabulated. Separate analyses were performed for people diagnosed with NHL and HL. We have also repeated the analysis in a subset of NHL after restricting to those with DLBCL.

Among NHL, for the evaluation of the association between HIV-infection status and risk of death we employed a manual staged strategy for the control of potential confounding: 1) Model B, which includes demographic variables (age, gender and calendar year of diagnosis of lymphoma; 2) Model C, including tumor-related characteristics such as the use of rituximab in the chemotherapy regimen and the standard IPI [age>60 y, stage III or IV, serum LDH> the upper limit of test as reported in the different center during the years of observation, ECOG performance status>1, more than 1 extranodal site]; 3) Model D, including all the variables in model B and C; and model E) which includes the same variables of model D but replacing standard IPI with age-adjusted IPI (stage III or IV, serum LDH> the upper limit of test as reported in the different center during the years of observation, ECOG performance status>1, more than 1 extranodal site), in order to reduce the bias due to a different age distribution among HIV-infected and HIV-uninfected patients groups. Moreover, we fitted a Cox regression model on a subset of HIV-L and nHIV-L matched (fuzzy matching) by calendar time (±0.5 year) and age (± 5 years) and including the same variables considered in model D (model F). The results of this analysis were similar to those of the standard regression adjustment but included only a small subset of fully matched individuals and were omitted in the Results. We tried to use propensity scores adjustment but because of the lack of overlap in distribution of the scores, adjustment was even less efficient than that obtained when matching for individual variables.

Similarly to the approach used for NHL, a similar sequential adjustment strategy has been used for people with HL: model B) including demographic variables such as age, gender and calendar year of lymphoma diagnosis; model C) including tumor-related factors such as treatment regimen (ABVD vs others) and stage of disease; model D) including all variables in model B and C; model F) including the same variables of model D in a matching analysis (Table A in S1 File). Of note, there is no model E in this group as it is only possible to control for age and staging separately.

## Results

A total of 1,331 patients (465 HIV-L, (34%) and 866 nHIV-L (65%) with lymphoma diagnosed between January 2000 and December 2013 contributed for a total of 5,765 person/years of follow up (PYFU). Demographic characteristics were reported in Table 1. Median year of lymphoma diagnosis was similar between groups (2008, IQR: 2005–2011 for HIV-L; 2008, IQR: 2004–2011 for nHIV-L). Median CD4+ cell count and HIV-RNA at diagnosis of lymphoma in HIV-L were 232/mmc (IQR 132–417) and 2.34 log_10_ copies/ml (IQR 1.69–4.74), respectively. Baseline tumour characteristics according to HIV status are also reported in Table 1.

**Table 1:** Baseline tumor characteristics according with type of lymphoma and HIV status.

	HIV-L	nHIV-L	P- value	total
***Non Hodgkin Lymphoma*, *n (%)***	321 (35)	588 (64)		909
Median Age, (IQR)	46 (39–52)	63(49–72)	<0.001	54 (44–68)
Female,	54 (17)	308 (52)	<0.001	362 (39.8)
HIV transmission route				
*IDU*	63 (21)			
*MSM*	55 (18)			
*Heterosexual*	74 (24)			
*Other/unknown*	114 (37)	588 (100)		
HbsAg+	23 (7)			
*Unknown*	88 (27)	588 (100)		
HCVAb+	80 (25)			
*Unknown*	52 (16)	588 (100)		
IPI* score (n = 549)				
*Low*	56 (17)	149 (25)	0.02	205 (23)
*intermediate*	98 (30)	183 (31)		281 (31)
High	24 (7)	39 (7)		63 (7)	
Histotype (n = 901)				
DLBCL**	202(65)	559 (95)	<0.001	761 (84)
*SNCCL* ***(Burkitt, *Burkitt-like*)	84 (27)	27 (5)		111 (12)
*IBL*°	21 (6)	2 (0.3)		23 (3)
*PBL*§	6 (2)	0 (0)		6 (1)
First line chemotherapy (n = 868)	300 (93)	551 (94)	<0.001	851 (94)
SNC°° prophylaxis (n = 607)	145 (45)	109 (18)	<0.001	254 (28)
CHOP+, CHOP-like++ regimens (n = 809)	230 (72)	419 (71)	0.001	649 (71)
Other regimens (n = 909)	82 (25)	116 (20)	0.04	198 (22)
Use of rituximab (n = 762)	172 (54)	387 (66)	0.001	599 (61)
***Hodgkin’s lymphoma*, *n (%)***	145 (32)	278 (32)		423
Median Age, (IQR)	46 (39–52)	63(49–72)	<0.001	54 (44–68)
Female,	16 (11)	140 (50)	<0.001	156 (37)
HIV transmission route				
*IDU*	23 (16.5)			
*MSM*	33 (24)			
*Heterosexual*	42 (30)			
*Other/unknown*	41 (29)	278 (100)		
HbsAg+	14 (10)			
*Unknown*	36 (25)	278 (100)		
HCVAb+	35 (24)			
*Unknown*	14 (10)	278 (100)		
Histotype (n = 422)				
*Nodular sclerosing*	32(22)	195 (70)	<0.001	227 (54)
*Mixed cellular*	51 (35)	15 (5)		66 (16)
*Lymphocyte-depleted*	4 (3)	5 (2)		9 (2)	
*Lymphocyte-rich*	7 (5)	15 (5)		22 (5)
*unspecified*	50 (34)	48 (17)		98 (23)
Ann Arbor stage (n = 405)				
*1*	66 (46)	86 (31)	0.02	152 (36)
*2*	32 (22)	127 (46)		159 (38)
*3*	36 (25)	58 (21)		94 (22)
*4*	50 (35)	69 (25)		119 (28)
First line chemotherapy (n = 419)	134 (93)	277 (99)	0.004	411 (97)
ABVD# (n = 392)	111 (96)	160 (58)	<0.001	271 (69)
VEBEP## (n = 359)	29 (20)	0	<0.001	29 (7)
BEACOPP### (347)	16 (11)	56 (20)	0.59	72 (17)

In parentheses after each variable, numbers of patients with available datum are reported, when applicable.

*International prognostic Index;

** Diffuse large B cell lymphoma;

***small non cleaved cell lymphoma;

° immunoblastic lymphoma;

^§^ plasmablastic lymphoma;

°°central nervous system;

^+^ cyclophosphamide, Adriamycin, vincristine, prednisone;

^++^ regimens containing at least cyclophosphamide,adriamycin;

^#^ doxorubicin, bleomycin+vinblastine, dacarbazine;

^##^ vincristine, epidoxorubicin, bleomycin, cyclophosphamide, etoposide, prednisone;

^###^ bleomycin, etoposide, Adriamycin, cyclophosphamide, vincristine, procarbazne, prednisone.

Among NHL, the distribution of histotypes was significantly different between the two study groups (p<0.001): DLBCL accounted for 95% of the total nHIV-L and for 65% of the HIV-L, while small non cleaved cells lymphomas (SNCCL) either Burkitt and Burkitt-like lymphoma, were reported in 5% and 27%, respectively. Of note, a significant lower proportion of use of rituximab-based regimens were reported in HIV-L (54%) vs. nHIV-L (66%) (p = 0.001; Table 1). Seventy-six HIV-negative patients and 1 HIV-positive patients underwent autologous hematopoietic stem cells transplantation (HASCT) as first line chemotherapy.

Similarly, the distribution of histotypes was significantly different between HIV-L and nHIV-L also in the HL subgroup (p<0.001): the nodular sclerosis variant was observed in 70% of nHIV-L vs. 22% of HIV-L, and mixed cellularity variant in 5% vs. 35% respectively (Table 1).

Among HIV-L, 125 patients (27%) were cART-naive at lymphoma diagnosis and started cART during chemotherapy at a median of 1 month after the diagnosis of lymphoma (IQR 0.4–3), while 292 patients (62%) developed lymphoma while already on cART for a median of 54 months (IQR 11–127); 45 patients (10%) never started cART during chemotherapy.

Of the 976 person who were still alive at the time of the analysis, 406 (42%) had their last visit more than 18 months before the date of freezing of the database (this percentage was 23% for the HIV-positive and 50% for the HIV-negative population, p = 0.0001) and 460 (47%) more than 12 months (30% vs. 55%, respectively, p = 0.0001). It is possible that after sometime in which HIV-negative participants have been in remission, follow-up in the hospital database is interrupted. This would lead to a violation of the non-informative censoring assumption and potential introduction of collision bias (censoring is associated with a lower risk of death). However, results where similar in a weighted Cox regression model with weights the inverse probability of censoring which should account for this potential bias (Table B in S1 File).

### Overall survival in whole population studied according to HIV status

Overall, 557 patients died (362 HIV-L and 195 nHIV-L). The 3-year overall probability of death was significantly higher in HIV-L group (34%, 95%CI 29–38%) vs. nHIV-L (18%, 95%CI 15–21%) [P<0.001]. 111 patients with HIV-L (85%) and 104 (75%) with nHIV-L died from lymphoma-related causes (progressive disease). In the HIV-L subgroup who ever started cART, the 3-year probability of death was comparable to that of all HIV-L patients (36%, 95%CI 31–42%).

### Overall survival in non- Hodgkin lymphoma (NHL)

**Fig 1:**
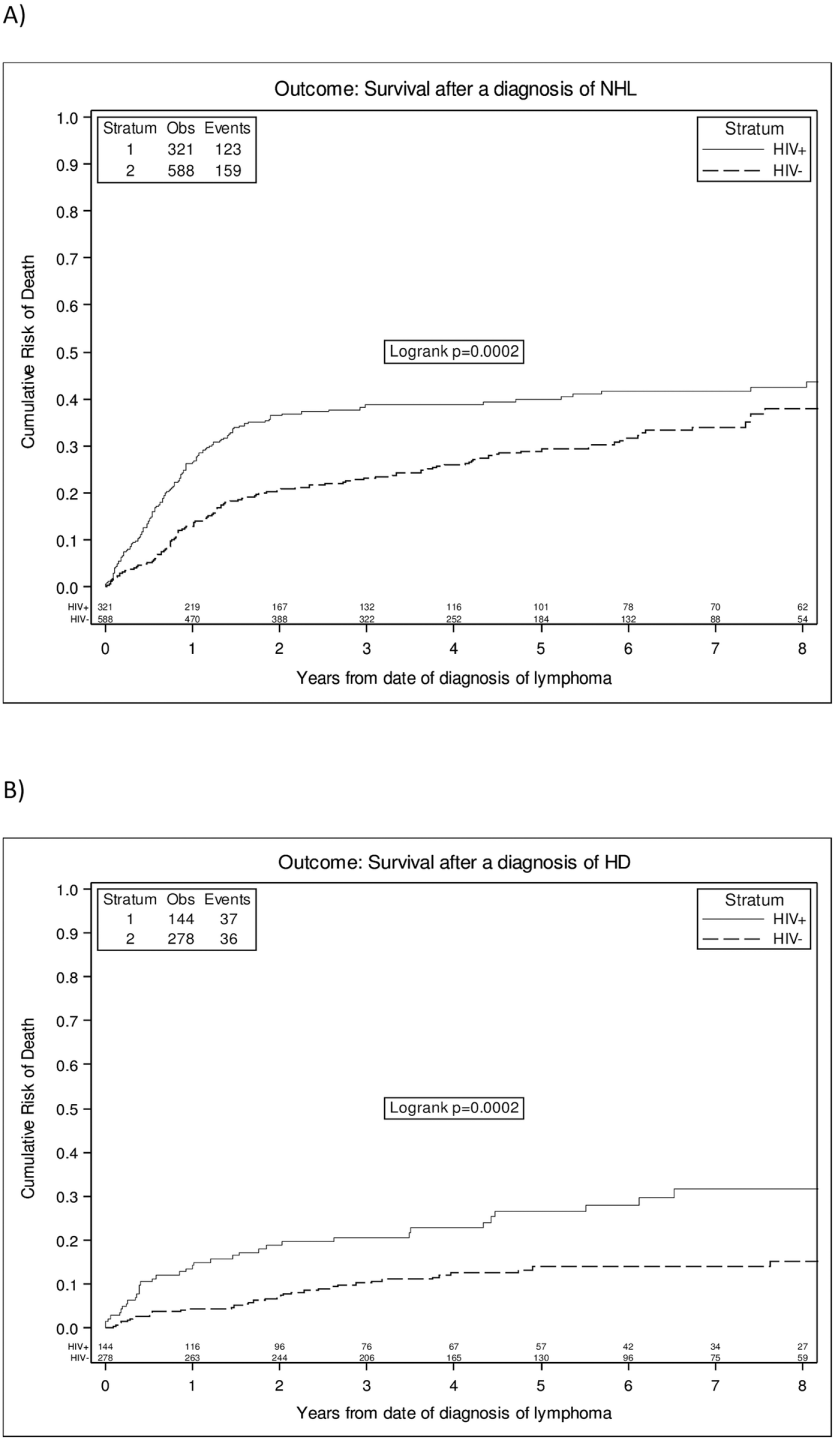
Crude estimates of survival according to HIV status in all NHL (a) and in HL (b).

In NHL subgroup, the overall 3-year probability of death was 38% (95%CI 33–44%) in HIV-L and 22% (95%CI 19–26%) in nHIV-L (p<0.001) (Fig 1A). HIV-NHL were independently associated with an increased risk of death in the fully adjusted model including age-adjusted IPI [Model E, adjusted hazard ratio (AHR) 1.69 (95%CI 1.26–2.26); p<0.001] (Table 2). Moreover, a further model including histotype as covariate showed similar results (AHR 1.66 (1.24–2.21) p<0.001). In contrast, when we controlled for standard IPI and age as separate factor, the risk in HIV-NHL vs. nHIV-NHL was somewhat attenuated and no longer significant (AHR = 1.26 (0.97–1.63), p = 0.08). Among other factors, older age (AHR 1.31 for 10 years older (1.19–1.45)); p<0.001] and a high IPI score (AHR for IPI 4–5: 6.95 (4.13–11.68), p<0.001) were both independently associated with an increased risk of death in contrast, female sex was associated with a reduced risk of death [AHR 0.74 vs. male (0.56–0.97)), p = 0.02] (Table 3).

**Table 2:** Relative hazard (HR) of death according with HIV status by different Cox regression models in n = 909 NHL.

	HR of HIV+ vs. HIV- (95% CI)	p-value
***Model A***		
Unadjusted	1.57 (1.24, 1.99)	< .001
***Model B***		
Adjusted for age, gender and calendar year of diagnosis	2.01 (1.51, 2.67)	< .001
***Model C***		
Adjusted for use of rituximab and standard IPI score	1.44 (1.13, 1.83)	0.01
***Model D***		
Adjusted for gender, calendar year of diagnosis, use of rituximab and standard IPI score	1.26 (0.97, 1.63)	0.08
***Model E***		
Adjusted for age, gender, calendar year of diagnosis, use of rituximab and age-adjusted IPI score	1.69 (1.26, 2.26)	<0.01

**Table 3:** Factors other than HIV-status associated with increased HR of death by multivariable Cox regression models according to type of lymphoma*.

	NHL		HL	
	Adjusted* RH (95% CI)	p-value	Adjusted* RH (95% CI)	p-value
***Age***				
per 10 years older	1.36 (1.19, 1.45)	<0.001	1.76 (1.47, 2.11)	< .001
***Gender***				
Female vs. Male	0.66 (0.52, 0.85)	0.001	1.28 (0.75, 2.19)	0.363
***Calendar year of diagnosis***				
per 5 more recent	1.02 (0.88, 1.18)	0.813	0.92 (0.66, 1.28)	0.604
***Ann Arbor Staging***	-			
***1***			1.00	
***2***			1.05 (0.34,3.21)	0.932
***3***			1.73 (0.57, 5.22)	0.329
***4***			1.94 (0.67, 5.61)	0.222
***IPI (age included)***		-		
0	1.00		-	
1–3	1.83 (1.43, 2.36)	< .001	-	
4–5	4.68 (3.04, 7.21)	< .001	-	
***Treatment with rituximab***				
Yes vs No	0.94 (0.70, 1.25)	0.65		
***Treatment with ABVD***	***-***		0.51 (0.27, 0.95)	0.033

* Table shows the results for the multivariable Model D for the HL and model E for the NHL group, respectively.

**Fig 2:**
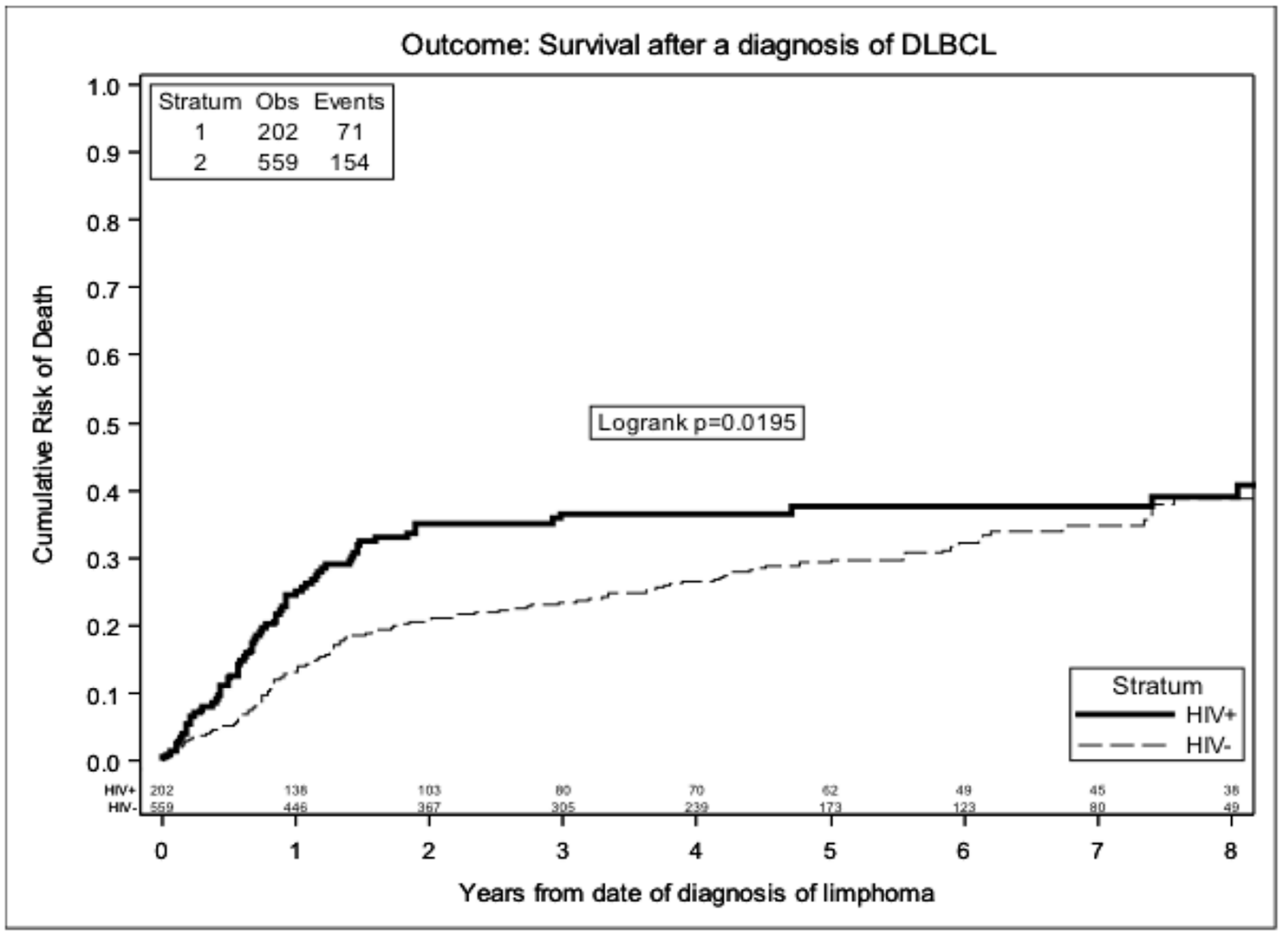
Crude estimates of survival according to HIV status in DLBCL.

When we restricted the analysis to the DLBCL subset of NHL, results were similar. The overall 3-year probability of death was 36% (95%CI 29–43%) in HIV-NHL and 22% (95%CI 19–26%) in nHIV-NHL (p<0.001) (Fig 2). Cox regression analysis restricted to DLBCL is shown in Table 4. After excluding patients who underwent HASCT results were similar (Table C in S1 File).

**Table 4:** Relative hazards from fitting a Cox regression model -DLBCL patients.

	HR of HIV+ vs. HIV- (95% CI)	p-value
***Model A***		
Unadjusted	1.40 (1.05, 1.86)	0.020
***Model B***		
Adjusted for age, gender and calendar year of diagnosis	1.79 (1.29, 2.47)	< .001
***Model C***		
Adjusted for use of rituximab and standard IPI score	1.30 (0.98, 1.73)	0.069
***Model D***		
Adjusted for gender, calendar year of diagnosis, use of rituximab and standard IPI score	1.14 (0.84, 1.54)	0.409
***Model E***		
Adjusted for age, gender, calendar year of diagnosis, use of rituximab and age-adjusted IPI score	1.50 (1.07, 2.09)	0.017

### Overall survival in Hodgkin lymphoma (HL)

The overall 3-year probability of death was 22% (15–29%) in HIV-HL and 10% (95%CI 6–13%) in nHIV-HL (log rank test p-value <0.0001). (Fig 1B). In this subgroup of patients, HIV-infection was associated with an even higher risk of death, with a more than two-fold increase in risk (AHR = 2.37 (95%CI 1.24–4.55; p = 0.01) when all variables were included in the models (model D) (Table 5) and even in a further model including histologic subtype (AHR = 2.38 (1.24, 4.57); p = 0.01). Among the other variables, older age [AHR for 10 years older 1.76 (1.47–2.11); p<0.001] and use of a non-ABVD chemotherapy regimen [AHR 0.51 (0.27–0.95); p = 0.03] resulted independently associated with the risk of death (Table 3).

**Table 5:** Relative hazard (HR) of death according with HIV status by different Cox regression models in n = 422 HL.

	HR of HIV+ vs. HIV- (95% CI)	p-value
***Model A***		
Unadjusted	2.32 (1.46, 3.67)	< .001
***Model B***		
Adjusted for age, gender and calendar year of diagnosis	2.41 (1.44, 4.05)	< .001
***Model C***		
Adjusted for use of ABVD and stage of disease	2.06 (1.10, 3.88)	0.025
***Model D***		
Adjusted for age, gender, calendar year of diagnosis, use of ABVD and stage of disease	2.37 (1.24, 4.55)	0.009

## Discussion

The results of this study indicate that, even in the cART era, the presence of HIV infection appears to remain a factor increasing the risk of death in patients with a diagnosis of Hodgkin lymphoma. For non- Hodgkin lymphoma, there was less evidence of an independent effect of HIV-status after controlling for key parameters such as lymphoma staging and use of rituximab.

Reports on the impact of HIV on survival in lymphoma patients have been so far somewhat inconsistent. Data coming from French Hospital Database on HIV infection showed that survival rates in HIV-infected individuals vs. HIV negatives in the time period of 2001–2004 were lower for patients with haematological malignancies but similar for patients with solid cancers. In particular, survival in patients with NHL was 49% in HIV-positive vs. 53% in HIV-negative, and 72% vs 79% in patients with HL, respectively [11]. A recent study on patients with a diagnosis of DLBCL, reported that, also in the cART era, this subtype of NHL shows more aggressive features in HIV-infected than those arising in the HIV uninfected population and has a poorer overall survival [7]. Nevertheless, cancer-free survival was comparable in HIV-positive and HIV-negative patients, suggesting that a higher frequency of death was not related to lymphoma but to other events in HIV-infected population [7]. In contrast, in a large series of DLBCL patients treated with rituximab-based regimens in United Kingdom, HIV-infected people had a significantly longer overall survival (5-year: 78% vs. 64%, p = 0.03) and disease-free survival (94% vs. 77%, p = 0.03), compared to HIV-uninfected people [8]. The authors concluded that HIV-positive patients diagnosed with DLBCL in the cART era have an excellent outcome, even better than that seen in the general population, when treated with standard immune-chemotherapy. Similarly, the use of standard chemotherapy has significantly improved the survival of HIV-infected people with Hodgkin lymphoma, approaching that of HIV-uninfected. A recent report from 404 patients with HL observed in France showed that, although high-risk features still predominate in HIV-associated HL, the prognosis of these patients, treated with cART as well as ABVD, has markedly improved in the modern cART era (2008–2014), and appears now to be similar to that of non-HIV-infected patients [12].

To our knowledge, our analysis was conducted on the largest database of people with NHL and HL lymphoma to date. One of the key aspects of analyses aiming at the evaluation of the association between HIV-infection status and OS is the choice of a suitable control. Specifically, if a significant difference between HIV-positive and HIV-negative individuals is detected, it is not simple to rule out whether this is due to HIV infection itself or to other factors, different in the HIV-L and nHIV-L populations, which are unmeasured or measured with error. Indeed in our case set, although we have carefully selected the HIV-negative control group for the same histological subtype (aggressive B cell lymphoma and HL) and time period of diagnosis as the HIV-infected participants, there were important measured differences in demographic, clinical and pathological features, according to HIV status. In particular, HIV-positive cases were younger and less frequently female. Histotype was less frequently DLBCL and more often SNCCL subtype in HIV-positive aggressive B cell lymphoma, and less frequently the nodular sclerosis variant and more often the mixed cellularity variant in HIV-positive HL. There were also differences in the treatment received. HIV-positive NHL received more frequently CHOP-like regimen, but less frequently a rituximab-containing regimen, and a proportion of HIV-positive HL received VEBEP regimen, while HIV-negative patients with advanced HL less than 60 years old received the BEACOPP regimen.

Potential confounding effect due to imbalances in demographic characteristics, histotype and treatment have been accounted for in a number of adjusted analysis. In fact, after controlling for differences in the prognostic IPI score and treatment with rituximab, the impact of HIV infection on survival was attenuated in NHL and DLBCL patients, although borderline significant. In contrast, even after adjustment for a similar set of confounding factors (i.e. age, calendar year, ABVD, stage), HIV-HL remained clearly independently associated with a higher risk of death compared to nHIV-HL.

Regarding the poorer survival in HIV-infected after a diagnosis of HL, we can only speculate on possible biological explanations. For example it is possible that HIV-positive HL are different from HIV-negative individuals for some unidentified or unmeasured risk factor for mortality, such as a difference in immune microenviroment [13]. The microenvironment plays a pivotal role in the pathogenesis of HL. The functional state of the T cells infiltrating the tumour lesions has an important prognostic impact in HIV-negative HL, and this functional state may be disturbed in the presence of HIV HL [14]. Further, a different role of EBV infection as driver of lymphomagenesis in the two cancers, as well as the different relationship with immunodeficiency, are alternative potential explanations for the different impact of HIV infection of survival of HL and NHL [14–16]. An additional explanation for the increased mortality among HIV-HL could reside in the eventual concomitant administration of vinblastine with boosted protease inhibitors. Indeed, this interaction (which could not be tested in our data) results in a significant increase of hematological toxicity, finally causing life-threatening infectious complications [17–18].

A large beneficial effect on survival has been seen after the inclusion of rituximab in chemotherapy regimens of patients with NHL, both in nHIV-L [19–20] and in HIV-L populations [21], and in our study a significant trend toward an increased use of rituximab has been observed in the last years of observation (Figure A in S1 File). In our analysis, HIV-L were less likely to use rituximab-based regimens than nHIV-L. Because of the strong association with the use of rituximab with longer survival (we found evidence for a 30% reduction in risk in those treated, although not strictly significant at 0.05 level), even after adjusting for calendar year, the difference in risk associated with HIV status was strongly attenuated after controlling for use of rituximab alone.

We have chosen to control for age using two different approaches: i) by including the standard IPI score (which simultaneously control for age) or ii) by including the age-adjusted IPI score and age as an additional separate factors. The association was more attenuated using approach i) perhaps because the standard IPI better controls for differences in age due to limited overlap in age distribution in HIV-positive (median age 46 years) and HIV-negative (median age 63 years) participants.

As most people were already receiving cART at the time of diagnosis or started within a month from the date of diagnosis (only 39 remained untreated over the duration of the study), it was difficult to evaluate whether any difference in survival between HIV-L and nHIV-L was attenuated by the use of cART. However, this was not the primary objective of our analysis, and when we restricted the analysis to the HIV-L who ever started cART, the 3-year probability of death was comparable to that of all HIV-L patients (Figures B and C in S1 File), suggesting that the any difference attributable to the comparison with untreated HIV-L is unlikely to be large. In addition cART use has been evaluated using an intention-to-treat principle (ever started cART vs. not) and potential interruption of cART during chemotherapy due to toxicities, drug-interactions or side effects have not been accounted for in our simple analysis. Nevertheless, the fact that the protective role of cART on the risk of developing clinical events appears to be not as strong in lymphomas as in other AIDS or non AIDS conditions is consistent with what found in other studies [22–24].

Our analysis has a number of limitations. First of all the choice of the control group: although it seemed the ideal group for HIV-infected who were diagnosed in UCSC in Rome, it might not be for the other HIV-L cohorts. However, the HIV-infected typically tend to move around clinical sites so an HIV-L resident in the north of Rome no necessarily would have been treated at UCSC. Moreover, the composition of the HIV-negative patient group is in line with epidemiological national data on aggressive lymphoma and HL. Second there could be differential assessment of key exposure variables. Although the same exact data collection questionnaire were used in HIV-L and nHIV-L it is conceivable that cancer-related variables were more accurately recorded in the notes of the nHIV-L population.

Third caveat and most important of all, unmeasured confounding factors cannot be ruled out. In particular, there was no measure of crucial life style factors associated with mortality such as smoking or alcohol intake, or of biological markers, such as EBV infection or other soluble factors potentially imbalanced between HIV-L and nHIV-L [25]. Moreover, immunological parameters such as CD4+ cell count were not collected for nHIV-L population making potentially immunologically not homogeneous the two studied populations; nevertheless, at our knowledge, in cohorts of nHIV-L these data are not systematically collected.

In conclusion, our cohort analysis shows a reduced survival in HIV-infected patients with HL compared to HIV-uninfected patients after adjusting for key potential confounders. In contrast, in people diagnosed with NHL, and particularly in those with DLBCL, only a slight trend toward a difference in survival attributable to HIV-infection was observed. This seemed to be mainly explained by more aggressive cancer presentation and reduced use of rituximab-based regimens in the HIV-infected population. These results are consistent with those of previous studies reporting similar survival following a diagnosis of HL according to HIV-status and underline the importance of an early diagnosis of neoplasia as well as of implementing use of standard therapy in HIV-infected patients with a diagnosis of lymphoma.

## Supporting information

S1 File Supplemental material. (DOCX)

S1 Data Study data set. (XLS)
