# Supplementary material for: Survival and predictors of death in people with HIV-associated lymphoma compared to those with a diagnosis of lymphoma in general population
Source: PLoS One. 2017 Oct 31;12(10):e0186549. doi: 10.1371/journal.pone.0186549 (PMC5663375; doi:10.1371/journal.pone.0186549)
Supplement: S1 File — (DOCX) [file pone.0186549.s001.docx]

**Table A in S1 File.** RH from fitting a Cox regression analysis in people matched using a propensity score

| Factor | HL lymphoma | | | NHL lymphoma | | |
| --- | --- | --- | --- | --- | --- | --- |
|  | Adjusted RH^*^ | 95% CI | p-value | Adjusted RH^*^ | 95% CI | p-value |
| HIV-positive vs. HIV negative | 1.80 | 0.52-6.20 | 0.36 | 1.18 | 0.75-1.87 | 0.48 |

^*^Model F): matched analysis

**Table B in S1 File.** RH from fitting a weighted Cox regression analysis with weights the inverse probability of censoring

| Factor | HL lymphoma | | | NHL lymphoma | | |
| --- | --- | --- | --- | --- | --- | --- |
|  | Adjusted RH^*^ | 95% CI | p-value | Adjusted RH^&^ | 95% CI | p-value |
| HIV-positive vs. HIV negative | 2.48 | 1.18-5.19 | 0.02 | 1.51 | 1.08-2.10 | 0.01 |

^*^Model D; ^&^Model E

**Table C in S1 File** – NHL lymphoma participants excluding pts who underwent AHSCT

|  | **Relative hazards of death-DLBCL patients** | |
| --- | --- | --- |
|  | **RH of HIV+ vs. HIV- (95% CI)** | **p-value** |
| ***Model A*** |  |  |
| Unadjusted | 1.33 (0.99, 1.77) | 0.056 |
| ***Model B*** |  |  |
| Adjusted for age, gender and calendar year of diagnosis | 1.81 (1.28, 2.55) | <.001 |
| ***Model C*** |  |  |
| Adjusted for use of rituximab and standard IPI score | 1.18 (0.88, 1.58) | 0.266 |
| ***Model D*** |  |  |
| Adjusted for gender, calendar year of diagnosis, use of rituximaband standard IPI score | 1.03 (0.75, 1.40) | 0.861 |
| ***Model E*** |  |  |
| Adjusted for age, gender, calendar year of diagnosis, use of rituximaband age-adjusted IPI score | 1.39 (0.97, 1.99) | 0.074 |
|  |  |  |

|  |  |  |
| --- | --- | --- |

**Figure A in S1 File** Percentage of rituximab use by calendar period of NHL diagnosis

| Started rituximab | 22 | 54 | 123 | 121 | 85 | 154 |
| --- | --- | --- | --- | --- | --- | --- |
| Total NHL | 157 | 145 | 172 | 140 | 102 | 193 |

**Figure B in S1 File** Survival KM estimates after a diagnosis of HL-lymphoma by HIV status after restricting to HIV-infected people who ever started ART

**Figure C in S1 File** Survival KM estimates after a diagnosis of NHL-lymphoma by HIV status after restricting to HIV-infected people who ever started ART
